# Socioeconomic status differs between breast cancer patients treated with mastectomy and breast conservation, and affects patient-reported preoperative information

**DOI:** 10.1007/s10549-019-05496-2

**Published:** 2019-11-18

**Authors:** A. Frisell, J. Lagergren, M. Halle, J. de Boniface

**Affiliations:** 1 Department of Molecular Medicine and Surgery, L1:00, Karolinska Institutet, 171 76 Stockholm, Sweden; 2 Department of Surgery, Capio St. Göran’s Hospital, Stockholm, Sweden; 3 Department of Reconstructive Plastic Surgery, Karolinska University Hospital, Stockholm, Sweden

**Keywords:** Breast-conserving surgery, Information, Socioeconomic status

## Abstract

**Purpose:**

Breast cancer treatment is reported to be influenced by socioeconomic status (SES). Few reports, however, stem from national, equality-based health care systems. The aim of this study was to analyse associations between SES, rates of breast-conserving surgery (BCS), patient-reported preoperative information and perceived involvement in Sweden.

**Methods:**

All women operated for primary breast cancer in Sweden in 2013 were included. Tumour and treatment data as well as socioeconomic data were retrieved from national registers. Postal questionnaires regarding preoperative information about breast-conserving options and perceived involvement in the decision-making process had previously been sent to all women receiving mastectomy.

**Results:**

Of 7735 women, 4604 (59.5%) received BCS. In addition to regional differences, independent predictors of BCS were being in the middle or higher age groups, having small tumours without clinically involved nodes, being born in Europe outside Sweden, having a higher education than primary school and an intermediate or high income per household. Women with smaller, clinically node-negative tumours felt more often involved in the surgical decision and informed about breast-conserving options (both *p* < 0.001). In addition, women who perceived that BCS was discussed as an alternative to mastectomy were more often in a partnership (*p *< 0.001), not born in Sweden (*p *= 0.035) and had an employment (*p *= 0.031).

**Conclusion:**

Socioeconomic factors are associated with surgical treatment even in a national health care system that is expected to offer all women the same standard of care. This should be taken into account and adapted to in preoperative counselling on surgical options in breast cancer.

## Introduction

The oncological equivalence between breast-conserving surgery (BCS) followed by adjuvant radiotherapy (RT) and mastectomy was first shown in large randomised trials conducted several decades ago [1, 2]. Despite a higher rate of local recurrences after BCS, survival rates were equal and thus, BCS became a valid option for the surgical treatment of early breast cancer. More recent retrospective studies, however, can no longer confirm an increased rate of local recurrences after BCS followed by adjuvant RT, and even point towards superior survival rates [3–5]. BCS increases postoperative satisfaction and leads to a better quality of life compared with mastectomy, with or without reconstruction [6]. Thus, current evidence strongly supports an increased use of BCS.

Patient and tumour characteristics, as well as surgeon and patient preference, are the main determinants when deciding on the type of surgical intervention [7]. Preoperative information regarding surgical treatment and patient-experienced participation in the decision-making process may vary considerably, yet have a significant impact on patients’ choice, post-decision regret, and patient satisfaction [8, 9]. Socioeconomic status (SES) is known to affect breast cancer treatment [10, 11] as well as perceived patient information and involvement in decision-making [12]. The majority of such reports come from the United States, where insurance issues and reimbursement patterns may potentially explain some of these observations.

The national average of immediate breast reconstruction (IBR) in Sweden is low in an international context at 8.5% in 2013; it has, however, been slowly increasing and has recently reached 14% [13]. The country´s sparse population with relatively long distances between centres may explain why regional differences are striking, with 32% in Stockholm and 8–11% in the Southern regions [13]. This discrepancy—despite national guidelines—has been addressed by our group previously [14, 15]. Here, we could show that regional differences were not—as claimed by many clinicians—due to differences in tumour characteristics and radiotherapy, but based on variations in preoperative patient information and involvement in the decision-making progress, as well as on socioeconomic status [14, 15].

The aim of this study was therefore to explore associations between surgical treatment, i.e. rates of breast conservation, SES and preoperative information as well as patient-perceived involvement in the Swedish setting where a public healthcare system aims to provide equal treatment options for all citizens.

## Patients and methods

This retrospective cohort study included all women operated for primary breast cancer in Sweden in 2013. Data on tumour and patient characteristics, surgery and postoperative treatment were received from the Swedish National Breast Cancer Register (NKBC). For a previous publication [14], all surviving patients from this cohort treated by mastectomy with or without immediate reconstruction had been sent a postal questionnaire up to 2 years after their surgery (during 2015). The questions included, amongst others, whether the patient had discussed breast-conserving options prior to mastectomy, whether the decision for mastectomy was taken by patient, surgeon or both together, and whether the patient felt involved in the decision-making process. At that time, no questionnaire was sent to patients receiving breast-conserving surgery due to the design of the original study. The response rate after one postal reminder was 76.3% (2217 of 2906).

An updated database extraction was requested from the Swedish National Breast Cancer Registry in 2016, and was subsequently completed with de-identified socioeconomic data from the Central Bureau of Statistics Sweden on all included patients. For patients operated for bilateral breast cancers, only one side was randomly selected. Variables received were family status, country of birth, education level, occupation, and disposable income per household as per year of interest (2013). The disposable income was classified into three groups by dividing the cohort into equal percentages. The highest level of education was divided into four groups according to the Swedish educational system: primary school, secondary school, post-secondary school ≤ 3 years, or post-secondary school > 3 years. The resulting database is registered and managed in accordance with the European General Data Protection Regulation (GDPR).

### Statistical analysis

Two groups were created for comparison: patients treated with breast-conserving surgery and patients treated with mastectomy with or without IBR. However, the IBR patients were significantly different to mastectomy patients in many aspects. Therefore, we first performed the analysis including IBR patients, and secondly additional analyses excluding IBR patients, in order to compensate for the similarities between BCS and IBR groups.

Categorical data are presented as numbers with their percentages, and continuous variables as median values with their range. For the comparison of categorical variables between the two groups, the Chi Square or Fisher’s exact tests, respectively, were used. Univariable binary logistic regression analysis was performed to study the association of tumour and patient characteristics as well as socioeconomic factors with the performance of breast-conserving surgery versus mastectomy. Subsequently, all factors were entered into a multivariable regression model. Results are presented as odds ratios (OR) with their respective 95% confidence intervals (CI).

Questionnaire results from mastectomy patients were selectively analysed concerning the questions “Did your surgeon discuss the option of breast-conserving surgery?” (Yes/Yes, partly/No), “Who took the decision to choose mastectomy?” (My choice, Surgeon’s choice, Both), and “Did you feel involved in the decision-making process to choose mastectomy?” (Yes/Yes, partly/No). For statistical analysis, the answers “Yes” and “Yes, partly”, and “My choice” and “Both”, were merged into one group each. Answers were first analysed in the entire mastectomy population and thereafter selectively in invasive breast cancer cases only, where two subgroups were compared: one with smaller tumours (cT1) that should have been technically feasible for breast-conserving surgery, and one with larger tumours (cT2-4).

All data analyses were performed using SPSS® version 24 (IBM, Armonk, New York, USA). Statistical significance was set at the 0.05 level for all analyses.

## Results

Overall, 7735 women were registered to have had surgery for primary breast cancer in 2013 in Sweden, of whom 4604 (59.5%) were operated with breast-conserving surgery (BCS) and 3131 (40.5%) with mastectomy. Of the latter group, 267 women (8.5%) had received immediate breast reconstruction (IBR). Due to the structure of the register, no data on delayed breast reconstruction were available.

Pre- and postoperative patient and tumour characteristics are shown in Table 1. As women receiving IBR may represent a patient population different to those receiving conventional mastectomy, additional analyses were performed excluding IBR patients from the mastectomy group. By that, the oldest age group (> 65 years) increased to 52.8% of the mastectomy cohort, while all listed factors retained their significant group differences.

**Table 1:** Patient and tumour characteristics for all women who underwent breast cancer surgery in Sweden in 2013 (*n* = 7735)

	Breast-conserving surgery (*n* = 4604)	Mastectomy with or without IBR (*n* = 3131)	*p*
Age (years)			< 0.001
≤ 40	148 (3.2)	193 (6.2)	
41–50	702 (15.2)	527 (16.8)	
51–65	1860 (40.4)	883 (28.2)	
> 65	1894 (41.2)	1528 (48.8)	
Preoperative clinical tumour stage			< 0.001
cTis (in situ only)	223 (4.8)	140 (4.5)	
cT1 (≤ 20 mm)	3687 (80.1)	1396 (44.6)	
cT2 (21–50 mm)	647 (14.1)	1223 (39.1)	
cT3 (> 50 mm)	23 (0.5)	290 (9.3)	
cT4	5 (0.1)	61 (1.9)	
Missing or unknown	19 (0.4)	21 (0.7)	
Preoperative node status			< 0.001*
cN0	4344 (94.4)	2534 (80.9)	
cN1	221 (4.8)	566 (18.1)	
Missing	39 (0.8)	31 (1.0)	
Postoperative invasive tumour size (mm)^a, c^	14 (0–140)	22 (0–245)	< 0.001**
Postoperative histopathological node status			< 0.001
Negative	3321 (72.1)	1534 (49.0)	
Positive	710 (15.4)	666 (21.3)	
Missing	573 (12.4)	931 (29.7)	
Invasiveness			< 0.001
In situ only	576 (12.5)	303 (9.6)	
Invasive	4022 (87.4)	2826 (90.3)	
Missing	6 (0.1)	2 (0.1)	
Presence of multifocality	376 (8.2)	747 (23.9)	< 0.001
Nottingham histological grade^a, b^			< 0.001
1	1112 (25.4)	372 (12.4)	
2	2038 (46.5)	1429 (47.8)	
3	1088 (24.8)	1053 (35.2)	
Missing	143 (3.3)	137 (4.6)	
Oestrogen receptor status^b^			< 0.001
Negative	418 (10.4)	474 (16.8)	
Positive	3542 (87.9)	2316 (81.9)	
Missing	68 (1.7)	38 (1.3)	
Progesterone receptor status^b^			< 0.001
Negative	848 (21.1)	841 (29.7)	
Positive	3102 (77.0)	1939 (68.6)	
Missing	78 (1.9)	48 (1.7)	
Her2/neu status^b^			< 0.001
Negative	3554 (88.2)	2299 (81.3)	
Positive	378 (9.4)	442 (15.6)	
Missing	96 (2.4)	87 (3.1)	
Proliferation (Ki-67 in %)^b, c^	18 (0–100)	25 (0–100)	< 0.001**

Values in parentheses are percentages unless indicated otherwise

*For all comparisons, Chi Square test, **Fisher’s exact test or Mann–Whitney U test were employed

^a^Excluding patients with neoadjuvant treatment

^b^Excluding patients with only in situ disease (DCIS)

^c^Values are median (range)

Group differences concerning socioeconomic background data are shown in Table 2. When looking more closely at differences between regions of own birth, 14.1% of women born in Sweden and 12.7% of women born in Europe outside Sweden, but only 9% of women born outside of Europe perceived the decision of mastectomy as their own or theirs together with the surgeon (*p *= 0.002). When again excluding IBR patients from the mastectomy group, however, the own birth country did not differ significantly between the groups (*p *= 0.162), while all other factors diverged even more strongly. In fact, IBR patients were most often married (61.1%), had least often a Swedish background (82.8%) and most often the highest level of education (35.7%), and were most often employed as clerks or civil servants (55.5%) with a high income per household (55.1%). These last four features were significantly different even from the BCS group (all *p *> 0.001), implicating that IBR patients represent a wealthier subgroup substantially different from conventional mastectomy patients.

**Table 2:** Socioeconomic status for women who underwent breast cancer surgery in Sweden in 2013 (n = 7735)

	Breast-conserving surgery (*n* = 4604)	Mastectomy, with or without IBR (*n* = 3131)	*p*
Family status			< 0.001
Partnership/married	2677 (58.1)	1651 (52.7)	
Single	1905 (41.4)	1454 (46.5)	
Missing	22 (0.5)	26 (0.8)	
Own birth country			0.042
Sweden	3954 (85.9)	2708 (86.5)	
Europe, not Sweden	459 (10.0)	269 (8.6)	
Outside of Europe	191 (4.1)	154 (4.9)	
Highest level of education			< 0.001
Primary school	888 (19.3)	871 (27.8)	
Secondary school	2026 (44.0)	1210 (38.6)	
Post-secondary school education, 3 years or less	665 (14.4)	390 (12.5)	
Post-secondary school education, more than 3 years	984 (21.4)	625 (20.0)	
Missing	41 (0.9)	35 (1.1)	
Occupation			< 0.001
Clerk/civil servant	1272 (27.6)	734 (23.4)	
Entrepreneur	173 (3.8)	98 (3.1)	
Labourer	761 (16.5)	421 (13.5)	
Unemployed/retired	2378 (51.7)	1838 (58.7)	
Missing	20 (0.4)	40 (1.3)	
Income per household			< 0.001
Low	1339 (29.1)	1230 (39.3)	
Middle	1625 (35.3)	936 (29.9)	
High	1628 (35.4)	955 (30.5)	
Missing	12 (0.3)	10 (0.3)	

*IBR* immediate breast reconstruction

Values in parentheses are percentages. For comparison of categorical variables, the Chi Square test was employed

Lower socioeconomic status was associated with larger clinical tumour size (*p *< 0.001 for all variables) as was being born outside Europe (median invasive tumour size 19 mm vs. 16 mm, *p *= 0.002). In the latter subgroup, axillary lymph nodes were significantly more often clinically positive (16% vs. 9.8 and 11.8%, respectively; *p *< 0.001).

Regional distributions of all variables and the significant variation of BCS rates are reflected in Table 3. Even though tumour characteristics differed between regions, no explanatory patterns for BCS variations could be discerned. As illustrated, Stockholm/Gotland clearly differed from the other regions in all socioeconomic factors.

**Table 3:** Regional variations of breast-conserving surgery, preoperative patient characteristics, tumour data and socioeconomic status regarding all women operated for primary breast cancer in Sweden 2013 (*N* = 7735)

Swedish healthcare region	North (*n* = 651)	Stockholm/Gotland (*n* = 1719)	South (*n* = 1529)	Southeast (*n* = 777)	Uppsala/Örebro (*n *= 1612)	West (*n* = 1447)	*p*
Breast-conserving surgery	420 (64.5)	1136 (66.1)	867 (56.7)	394 (50.7)	951 (59.0)	836 (57.8)	< 0.001
Preoperative clinical T stage							< 0.001
In situ only	30 (4.6)	68 (4.0)	119 (7.8)	17 (2.2)	61 (3.8)	68 (4.7)	
cT1	446 (68.5)	1145 (66.6)	984 (64.4)	537 (69.1)	1011 (62.7)	960 (66.3)	
cT2	141 (21.7)	399 (23.2)	363 (23.7)	177 (22.8)	434 (26.9)	356 (24.6)	
cT3	20 (3.1)	88 (5.1)	38 (2.5)	37 (4.8)	82 (5.1)	48 (3.3)	
cT4	11 (1.7)	13 (0.8)	14 (0.9)	4 (0.5	17 (1.1)	7 (0.5)	
Missing	3 (0.5)	6 (0.3)	11 (0.7)	5 (0.6)	7 (0.4)	8 (0.6)	
Preoperative clinical N stage							0.002
cN0	571 (87.7)	1568 (91.2)	1345 (88.0)	691 (88.9)	1422 (88.2)	1281 (88.5)	
cN1	67 (10.2)	144 (8.4)	168 (11.0)	81 (10.4)	169 (10.5)	158 (10.9)	
Missing	13 (2.0)	7 (0.4)	16 (1.0)	5 (0.6)	21 (1.3)	8 (0.6)	
Age at surgery*	64 (28-97)	63 (21–96)	63 (26–94)	64 (21–93)	64 (22–94)	63 (26–97)	< 0.001**
Family status							0.015
Partnership/married	372 (57.1)	907 (52.8)	862 (56.4)	477 (61.4)	881 (54.7)	829 (57.3)	
Single	272 (41.8)	802 (46.7)	659 (43.1)	295 (38.0)	721 (44.7)	610 (42.2)	
Missing	7 (1.1)	10 (0.6)	8 (0.5)	5 (0.6)	10 (0.6)	8 (0.6)	
Own birth country							< 0.001
Sweden	585 (89.9)	1357 (78.9)	1339 (87.6)	698 (89.8)	1416 (87.8)	1267 (87.6)	
Europe, not Sweden	50 (7.7)	222 (12.9)	151 (9.9)	55 (7.1)	138 (8.6)	112 (7.7)	
Outside of Europe	16 (2.5)	140 (8.1)	39 (2.6)	24 (3.1)	58 (3.6)	68 (4.7)	
Highest level of education							< 0.001
Primary school	130 (20.0)	302 (17.6)	377 (24.7)	205 (26.4)	415 (25.7)	330 (22.8)	
Secondary school	290 (44.5)	647 (37.6)	673 (44.0)	334 (43.0)	713 (44.2)	579 (40.0)	
Postsecondary, 3 years or less	80 (12.3)	274 (15.9)	186 (12.2)	100 (12.9)	205 (12.7)	210 (14.5)	
Postsecondary, more than 3 years	146 (22.4)	470 (27.3)	281 (18.4)	133 (17.1)	265 (16.4)	314 (21.7)	
Missing	5 (0.8)	26 (1.5)	12 (0.8)	5 (0.6)	14 (0.9)	14 (1.0)	
Occupation							< 0.001
Clerk/civil servant	178 (27.3)	555 (32.3)	364 (23.8)	181 (23.3)	350 (21.7)	378 (26.1)	
Entrepreneur	31 (4.8)	58 (3.4)	42 (2.7)	23 (3.0)	64 (4.0)	53 (3.7)	
Labourer	100 (15.4)	204 (11.9)	259 (16.9)	129 (16.6)	257 (15.9)	233 (16.1)	
Unemployed/retired	337 (51.8)	891 (51.8)	859 (56.1)	438 (56.4)	924 (57.3)	768 (53.1)	
Missing	5 (0.8)	11 (0.6)	6 (0.4)	6 (0.8)	17 (1.1)	15 (1.0)	
Income per household							< 0.001
Low	234 (35.9)	482 (28.0)	519 (33.9)	250 (32.2)	587 (36.4)	497 (34.3)	
Average	222 (34.1)	539 (31.4)	530 (34.7)	277 (35.6)	557 (34.6)	436 (30.1)	
High	193 (29.6)	692 (40.3)	477 (31.2)	247 (31.8)	463 (28.7)	511 (35.3)	
Missing	2 (0.3)	6 (0.3)	3 (0.2)	3 (0.4)	5 (0.3)	3 (0.2)	

Values in parentheses are percentages unless indicated otherwise: *values are median (range), ** Kruskal–Wallis test. For comparison of categorical variables, the Chi Square test was employed

### Factors affecting BCS rates

Independent predictors for undergoing BCS are shown in Table 4. When running the same multivariable regression analysis excluding those women having received an IBR, also the oldest, together with the youngest age group showed the lowest probability to receive BCS, and having the highest level of education did no longer act as an independent predictor of BCS (OR 1.18, 95% CI 0.98–1.41). Living in the Stockholm/Gotland region resulted in a significantly increased likelihood of BCS compared to the reference region North (OR 1.39, 95% CI 1.10–1.76).

**Table 4:** Univariable and multivariable binary logistic regression analyses of clinical and socioeconomic factors with performance of breast-conserving surgery as opposed to mastectomy (with or without IBR) as the binary endpoint

	Univariable		Multivariable	
	Odds ratio	*p*	Odds ratio	*p*
Age (years)				
≤ 40	1.00 (reference)			
41–50	1.74 (1.36–2.21)	< 0.001	1.58 (1.19–2.10)	0.001
51–65	2.75 (2.19–3.45)	< 0.001	2.36 (1.80–3.09)	< 0.001
> 65	1.62 (1.29-2.02)	< 0.001	1.74 (1.30–2.33)	< 0.001
Preoperative clinical tumour stage				
cTis (in situ only)	1.00 (reference)			
cT1 (≤ 20 mm)	1.66 (1.33–2.07)	< 0.001	1.87 (1.49–2.35)	< 0.001
cT2 (21–50 mm)	0.33 (0.26–0.42)	< 0.001	0.42 (0.33–0.54)	< 0.001
cT3 (> 50 mm)	0.05 (0.03–0.08)	< 0.001	0.07 (0.04–0.11)	< 0.001
cT4	0.05 (0.02–0.13)	< 0.001	0.10 (0.04–0.27)	< 0.001
Preoperative node status				
cN0	1.00 (reference)			
cN1	0.23 (0.19–0.27)	< 0.001	0.40 (0.33–0.48)	< 0.001
Family status				
Partnership/married	1.00 (reference)			
Single	0.81 (0.74–0.89)	< 0.001	1.01 (0.87–1.17)	0.901
Own birth country				
Sweden	1.00 (reference)			
Europe, not Sweden	1.17 (1.00–1.37)	0.054	1.38 (1.14–1.67)	0.001
Outside of Europe	0.85 (0.68–1.06)	0.142	1.23 (0.93–1.61)	0.144
Highest level of education				
Primary school	1.00 (reference)			
Secondary school	1.64 (1.46–1.85)	< 0.001	1.33 (1.16–1.53)	< 0.001
Post-secondary school education, 3 years or less	1.67 (1.43–1.96)	< 0.001	1.31 (1.10–1.59)	0.004
Post-secondary school education, more than 3 years	1.54 (1.35–1.77)	< 0.001	1.20 (1.00–1.42)	0.044
Occupation				
Clerk/civil servant	1.00 (reference)			
Entrepreneur	1.02 (0.78–1.33)	0.891	1.03 (0.76–1.40)	0.831
Labourer	1.04 (0.90–1.21)	0.581	1.11 (0.93–1.34)	0.256
Unemployed/retired	0.75 (0.67–0.83)	< 0.001	0.95 (0.79–1.13)	0.539
Income per household				
Low	1.00 (reference)			
Middle	1.60 (1.43–1.78)	< 0.001	1.39 (1.19–1.62)	< 0.001
High	1.57 (1.40–1.75)	< 0.001	1.29 (1.05–1.58)	0.014
Region				
North	1.00 (reference)			
Stockholm/Gotland	1.07 (0.89–1.30)	0.473	1.05 (0.84–1.31)	0.660
South	0.72 (0.60–0.87)	0.001	0.67 (0.54–0.83)	< 0.001
Southeast	0.57 (0.46–0.70)	< 0.001	0.48 (0.38–0.62)	< 0.001
Uppsala/Örebro	0.79 (0.66–0.96)	0.015	0.82 (0.66–1.02)	0.070
West	0.75 (0.62–0.91)	0.004	0.71 (0.57–0.88)	0.002

Values in parenthesis are 95% confidence intervals

### Patient-reported received information about BCS and patient involvement

As questionnaires regarding perceived patient information and involvement had only been sent to those being operated by mastectomy in our previous study, these women’s questionnaire results were selectively analysed. Women stating that the decision to have a mastectomy was their own or theirs together with their physician were older (*p *< 0.001), resided more often in the region North (*p *= 0.033), had more often smaller tumours (cT1, *p *< 0.001) without clinical lymph node involvement (*p *< 0.001), and were less often registered as working as labourers (*p *< 0.001). Those who reported having felt involved in the mastectomy decision were significantly older (*p *= 0.034) and had smaller tumours with clinically negative lymph nodes (both *p *= 0.001). Those women who reported that breast-conserving surgery was discussed as an alternative to mastectomy did not differ in age or region of residence, but had smaller tumours (*p *< 0.001) with clinically negative lymph nodes (*p *< 0.001), were more often in a partnership (*p *< 0.001), not born in Sweden (*p *= 0.035) and had an employment (*p *= 0.031). A tendency to have a higher income when reporting that breast-conserving surgery had been discussed was not statistically significant (*p *= 0.051).

When selectively analysing women with clinically smaller tumours (cT1) who should have been technically feasible candidates for BCS, rates of preoperative information on BCS and perceived involvement were rather low and varied significantly in different health care regions (Table 5).

**Table 5:** Patient-reported preoperative information about breast-conserving surgery and perceived involvement in surgical decision among women treated with mastectomy (with or without IBR) in each Swedish healthcare region

All ages	North	Stockholm/Gotland	South	Southeast	Uppsala/Örebro	West	*p*
Did your surgeon discuss the option of breast-conserving surgery?^a^							
cT1	60 (80.0)	83 (53.5)	115 (54.8)	95 (58.6)	100 (55.6)	121 (61.4)	0.002
cT2–4	23 (39.7)	90 (43.5)	78 (42.9)	36 (35.0)	88 (39.8)	70 (38.7)	0.730
Who took the decision to choose mastectomy?^b^							
cT1	56 (76.7)	94 (60.3)	138 (63.9)	110 (66.3)	109 (58.9)	130 (64.0)	0.132
cT2–4	37 (62.7)	124 (59.3)	91 (47.9)	58 (53.7)	122 (52.8)	95 (51.6)	0.182
Did you feel involved in the decision-making process to choose mastectomy?^a^							
cT1	66 (90.4)	123 (78.8)	179 (81.7)	149 (90.0)	163 (87.6)	168 (82.4)	0.014
cT2–4	49 (81.7)	175 (82.9)	145 (76.7)	87 (82.1)	186 (80.2)	150 (80.6)	0.737

^a^Values are the number of women who answered “Yes” or “Yes, partly” to each question, with percentages in parentheses

^b^Values are the number of women who answered “My choice” and “Both”, i.e. patient’s and surgeon’s choice, with percentages in parentheses. Patients with in situ disease only were excluded

## Discussion

Socioeconomic factors were significantly associated with BCS rates, even after adjusting for tumour and patient characteristics. As expected, women receiving BCS had a clinically lower tumour stage and clinically uninvolved lymph nodes, but were also overrepresented in the middle age groups, were born in Europe, had a higher education and a higher family income. Among women with the lowest clinical tumour stage (cT1) who received mastectomy, there were significant regional variations in patient-reported preoperative information regarding BCS and perceived own involvement, factors that were additionally associated with socioeconomic factors.

Our findings align with earlier observations showing associations between higher SES and increased BCS rates [10, 11, 14]. We could also confirm that women with lower SES present with a higher tumour stage [17], which may further influence the choice of mastectomy. Interestingly, patients receiving IBR were a highly selected, socioeconomically strong group. A potential association between the implementation of oncoplastic techniques, known to increase BCS rates, and rates of breast reconstruction may explain why BCS rates were highest in Stockholm once the subgroup of IBR patients was excluded from multivariable analyses. Not all units operating breast cancer patients are registered Breast Units according to European Society of Breast Cancer Specialists by means of the involvement of plastic surgeons, who are most commonly affiliated to university. However, surgeons treating breast cancer patients are specifically trained breast surgeons, coming from a background of general surgery.

The distance to travel to the nearest health provider and individual life circumstances may affect the ability or willingness to comply with adjuvant radiotherapy that is an integral part of breast conservation [16, 17]. Jacobs et al. showed that a longer distance from the radiation treatment was associated with lower rates of BCS [18]. Since radiotherapy requires frequent hospital visits, the inconvenience of travel and potentially temporary accommodation might play a significant role when choosing the surgical procedure [19]. Our results, however, showed that the North region, with the longest distances to health facilities, had the second highest BCS rate, which might be explained by a high rate of preoperative information.

When patients do not perceive being informed about breast-conserving options it can be due to the informing part (the surgeon, the breast nurse or other member from the multidisciplinary team) or the receiving part (the patient and her family/friends), or a combination of both. Low satisfaction with preoperative information regarding the surgical breast cancer treatment is associated with increased postoperative regret and anxiety [9]. The role of SES in this context needs to be debated; if women with a lower SES do not feel informed about the option of BCS, potential obstacles need to be identified and additional support strategies implemented. Whelan et al. showed that standardised information strategies regarding BCS led to a higher knowledge about the treatment options and increased satisfaction with the decision-making [20]. Presenting information in written, oral and visual form can also improve patient knowledge [21]. The use of repetition and take-home information about surgical choices which the patient may think about when less distressed may further improve understanding of available treatment options [22]. Measures such as these have been taken in Sweden (e.g. national information leaflets, individually assigned breast contact nurses and locally assembled information folders) since the studied year of inclusion, and a comparative analysis should be performed to evaluate the impact on the perceived information status of the patient.

Our results need to be interpreted in the light of some limitations. First, there is always a risk of recall bias in any retrospective study [23], as the women in this study received the questionnaires up to 2 years after their surgical treatment. Second, we had no information regarding hereditary breast cancer, which may affect the mastectomy rate especially among younger women. Third, studied BCS rates stem from the year 2013 which is 6 years ago; updated national reports, however, still demonstrate regional differences despite a national trend towards increased BCS rates [24]. Fourth, the regional lack of in-house plastic surgery services has a significant negative impact on IBR and patient information rates [14]. The main strengths of our study are the high coverage and validity of two nationwide population-based registers with detailed tumour and socioeconomic data [25, 26], and the fact that economical and reimbursement differences should not impact results due to the nature of the Swedish general health care system, where all breast cancer patients are operated within the public healthcare where the economic implications of treatment options are negligible as a confounder to SES.

In conclusion, this study provides new information regarding socioeconomic factors’ association with BCS rates, patient information and involvement in the decision-making process in a national system that should be providing equal health care to all individuals. Our results confirm that socioeconomic background should be taken into account in preoperative counselling.

## References

[1] Veronesi U (2002). Breast-conserving surgery with radical mastectomy. N Engl J Med.

[2] Fisher B (2002). Twenty-year follow-up of a randomized trial comparing total mastectomy, lumpectomy, and lumpectomy plus irradiation for the treatment of invasive breast cancer. N Engl J Med.

[3] de Boniface J (2017). Survival and axillary recurrence following sentinel node-positive breast cancer without completion axillary lymph node dissection: the randomized controlled SENOMAC trial. BMC Cancer.

[4] van Maaren MC (2016). 10 year survival after breast-conserving surgery plus radiotherapy compared with mastectomy in early breast cancer in the Netherlands: a population-based study. Lancet Oncol.

[5] Hofvind S, Holen A, Aas T, Roman M, Sebuødegård S, Akslen LA (2015). Women treated with breast conserving surgery do better than those with mastectomy independent of detection mode, prognostic and predictive tumor characteristics. Eur J Surg Oncol.

[6] Al-Ghazal SK, Fallowfield L, Blamey RW (2016). Comparison of psychological aspects and patient satisfaction following breast conserving surgery, simple mastectomy and breast reconstruction. Eur J Cancer.

[7] Molenaar S (2004). Predictors of patients’ choices for breast-conserving therapy or mastectomy: A prospective study. Br J Cancer.

[8] Sheehan JB, Sherman KA (2007). Association of information satisfaction, psychological distress and monitoring coping style with post-decision regret following breast reconstruction. Psychooncology.

[9] Caldon LJM (2011). Why do hospital mastectomy rates vary Differences in the decision-making experiences of women with breast cancer. Br J Cancer.

[10] Gu J, Groot G, Boden C, Busch A, Holtslander L, Lim H (2018). Review of factors influencing women’s choice of mastectomy versus breast conserving therapy in early stage breast cancer: a systematic review. Clin. Breast Cancer.

[11] Ridao-López M, García-Armesto S, Abadía-Taira B, Peiró-Moreno S, Bernal-Delgado E (2011). Income level and regional policies, underlying factors associated with unwarranted variations in conservative breast cancer surgery in Spain. BMC Cancer.

[12] Dahlbäck C, Manjer J, Rehn M, Ringberg A (2017). Patients undergoing breast-conserving surgery can benefit from the opportunity to participate in choosing their surgical technique. World J Surg.

[13] Bröstcancerregistret N (2018) Rapport från nationella sammanfattning bröstcancerregistret

[14] Frisell A, Lagergren J, de Boniface J (2016). National study of the impact of patient information and involvement in decision-making on immediate breast reconstruction rates. Br J Surg.

[15] Frisell A, Lagergren J, Halle M, de Boniface J Socioeconomic status influences immediate breast reconstruction rates, patient information and involvement in surgical decision-making. Submitted manuscript 10.1002/bjs5.50260 PMC7093785 32003544

[16] Mac Bride MB (2013). Factors associated with surgical decision making in women with early stage breast cancer: a literature review. J Women’s Health.

[17] Byers TE et al (2008) The impact of socioeconomic status on survival after cancer in the United States. American Cancer Society, pp 582–591 10.1002/cncr.23567 18613122

[18] Jacobs LK, Kelley KA, Rosson GD, Detrani ME, Chang DC (2008). Disparities in urban and rural mastectomy populations: the effects of patient- and county-level factors on likelihood of receipt of mastectomy. Ann Surg Oncol.

[19] Nattinger AB, Kneusel RT, Hoffmann RG, Gilligan MA (2001). Relationship of distance from a radiotherapy facility and initial breast cancer treatment. J Natl Cancer Inst.

[20] Trial AR (2004). Effect of a decision aid on knowledge and treatment decision making for breast cancer surgery. JAMA.

[21] Brown FF, Lewis JW, Harcleroad (1969). AV instruction technology, media, and methods.

[22] Falvo DR (1994). Effective patient education. A guide to increased compliance.

[23] Tourangeau R, Rips LJ, Rasinski K (2000). The psychology of survey response.

[24] Årsrapport 2018 från Nationella Bröstcancerregistret. no. September, 2018

[25] No title https://statistik.incanet.se/brostcancer/

[26] Projektrapport Validering av Nationellt kvalitetsregister för bröstcancer. 2015

